# Photocatalytic Reaction in Aqueous Suspension: FTIR Spectroscopy with Attenuated Total Reflection in Diamonds

**DOI:** 10.1021/acsomega.3c04330

**Published:** 2023-09-08

**Authors:** Zhebin Fu, Hiroshi Onishi

**Affiliations:** † Department of Chemistry, School of Science, Kobe University, Kobe, Hyogo 657-8501, Japan; ‡ Research Institute for Integrated Science, Kanagawa University, Yokohama, Kanagawa 221-8686, Japan; § Research Center for Membrane and Film Technology, Kobe University, Kobe 657-8501, Japan; ∥ Division of Advanced Molecular Science, Institute for Molecular Science, Okazaki, Aichi 444-8585, Japan

## Abstract

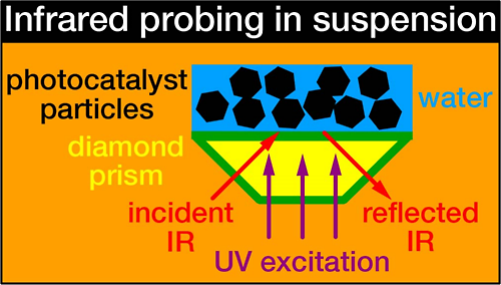

The photocatalytic conversion of an organic compound on rutile nanoparticles dispersed in aqueous solutions was characterized by infrared absorption spectroscopy. A diamond prism for total reflection of infrared light provided convenient and reliable access to the absorption spectrum of adsorbed chemical species photocatalytically converted under ultraviolet light irradiation. Pivalic acid, a reactant to be decarboxylated by hole capture, was dissolved in water at concentrations of 100–3 mmol L^–1^ and exhibited vibrational bands of 0.01–0.001 absorbance in the 1500–1100 cm^–1^ wave number region. When rutile particles were suspended in the solutions, dissociative adsorption leading to the formation of pivalate anions on the particles was detected in vibrational spectra. The adsorbed pivalate anions decomposed by ultraviolet light irradiation through the prism, releasing CO_2_. In an anaerobic atmosphere, the excited electrons were accommodated in the particles as small polarons, resulting in an optical absorption centered at 7000 cm^–1^. Conversely, in an aerobic atmosphere, the electrons were transferred to the surrounding atmosphere, eliminating the polaron-induced absorption. This study demonstrates the feasibility of infrared absorption spectroscopy for operando monitoring of vibrational and electronic transitions, enabling the tracking of photochemical reactions at liquid–solid interfaces.

## 1 Introduction

Research on materials conversion over semiconductor photocatalysts is being conducted worldwide. Downhill reactions, where the Gibbs free energy decreases during the conversion of reactants to products, have been successfully integrated into our society.^1^ Artificial photosynthesis, a category of uphill reactions involving the oxidation of water, is being developed for societal implementation in the near future.^2^ In addition, intensive fundamental studies are being conducted to uncover new scientific discoveries related to light-driven efficient conversion of materials.^3^

Operando characterization of semiconductor photocatalysts is being pursued to both accelerate implementation and deepen our intellectual understanding. Optical spectroscopy provides a valuable approach to the study of electronic transitions in photocatalysts. In particular, the transmission absorption of infrared (IR) light has been used to characterize the electronically excited states of photocatalysts in vacuum or vapor environments.^4−6^ The application of IR absorption spectroscopy should be extended to include photocatalysts suspended in aqueous solutions, where the majority of photocatalytic reactions occur. However, this is challenging. Attenuated total reflection (ATR) in a Si or ZnSe prism is used to direct IR light to the photocatalyst suspended in aqueous solution. Passing ultraviolet (UV) light through the prism to excite the photocatalyst is not feasible. Using UV illumination above the prism is also problematic because the light is absorbed by photocatalyst particles on the surface of the suspension. Particles probed by IR light at the bottom of the suspension are insufficiently excited.

We demonstrate that a diamond prism is effective in providing IR light for probing and UV light for excitation through the prism, as illustrated in the TOC graphic. Infrared absorption of excited electrons that have not yet recombined with holes has been observed in suspended particles of NaTaO_3_,^7^ anatase TiO_2_,^8^ and rutile TiO_2_.^8^ In the current study, we further utilize IR spectroscopy with the diamond prism to simultaneously detect vibrational absorption of molecular species and electronic absorption of excited electrons. The feasibility of the method demonstrated in this research is significant for the operando characterization of photochemical reactions at liquid–solid interfaces.

## 2 Reactions of Interest

Our target reaction is the degradation of pivalic acid, also known as trimethylacetic acid, on rutile TiO_2_ particles. The photocatalytic degradation of this compound was studied on atomically flat (110)-oriented rutile wafers. The (110) truncation of rutile has been extensively studied as a prototype for atomically flat truncation of metal oxides.^9−14^ When a (110)-oriented wafer is exposed to pivalic acid vapor, a densely packed monolayer of pivalate anions forms at room temperature, with each pivalate anion bridging two Ti^4+^ cations exposed on the surface. The acid proton is transferred to an oxygen anion protruding from the surface,^15^ as described by the following formula.
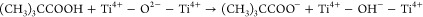
1

The pivalate anion captures a photoexcited hole and decomposes to a *t*-butyl radical, releasing a CO_2_ molecule into the gas phase.^16^
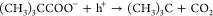
2

The *t*-butyl radical undergoes a β-hydride elimination to produce isobutene with a small amount of isobutane. Protons that were released during the dissociative adsorption of pivalic acid in formula 1 combined with photoexcited electrons to eventually produce water, when O_2_ is present in the atmosphere.^17^
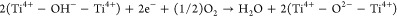
3

In the absence of oxygen, the electrons reduce the surface Ti cations adjacent to the protons from a 4+ to a 3+ state.
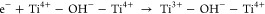
4

We hypothesized that hole-induced decarboxylation of pivalate would occur on rutile particles suspended in aqueous solutions of pivalic acid.

## 3 Experimental Section

A diamond prism with an isosceles trapezoidal shape and a circular reflection plane with a diameter of 1.8 mm was assembled with an LED (M365L3, Thorlabs). The incident angle of the IR light, θ, was fixed at 45° relative to the normal of the reflection plane. UV light with a center wavelength of 365 nm was passed through the prism and focused on the reflection plane. The optical characteristics of the prism assembly are described in the Supporting Information of ref (7).

A rutile TiO_2_ photocatalyst (JRC-TIO-6) was provided by the Catalysis Society of Japan. The photocatalyst particles were suspended in an aqueous solution of pivalic acid (>99.0%, Tokyo Chemical Industry, P0461) and placed on the reflection plane at RT. The concentrations of the solutions were adjusted to 102, 51, 26, 13, 6, and 3 mmol L^–1^, which are 1/2, 1/4, 1/8, 1/16, 1/32, and 1/64 of the solubility.^18^ The depth of IR light penetration into water, *d*, was approximated to be 0.76 μm at a wavenumber of 2000 cm^–1^ using the following formula
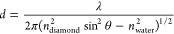
5where λ is the wavelength of IR light and the refractive index of diamond, *n*_diamond_, is 2.38 at 2000 cm^–1^. The refractive index of water, *n*_water_, was not available at 2000 cm^–1^ and was assumed to be 1.32, corresponding to the index at 10,000 cm^–1^. The refractive index of the suspensions is greater than *n*_water_ to increase *d* accordingly because rutile has an index of 2.22 at 2000 cm^–1^.^19^

The prism assembly was installed in an Fourier transform infrared (FTIR) spectrometer (FT/IR-6600, Jasco). Inside the spectrometer the assembly was exposed to either air or N_2_ gas (99.995%, Tomoe) to make the suspension anaerobic if necessary. The acquisition time for each spectrum was 19 s with a wavenumber resolution of 8 cm^–1^. In addition to the UV light used to excite the photocatalyst, the reflection plane was irradiated with He–Ne laser light (wavelength: 633 nm) for interferometer calibration.

## 4 Results and Discussion

### 4.1 IR Absorption of Condensed Pivalic Acid and Solution

**Figure 1:**
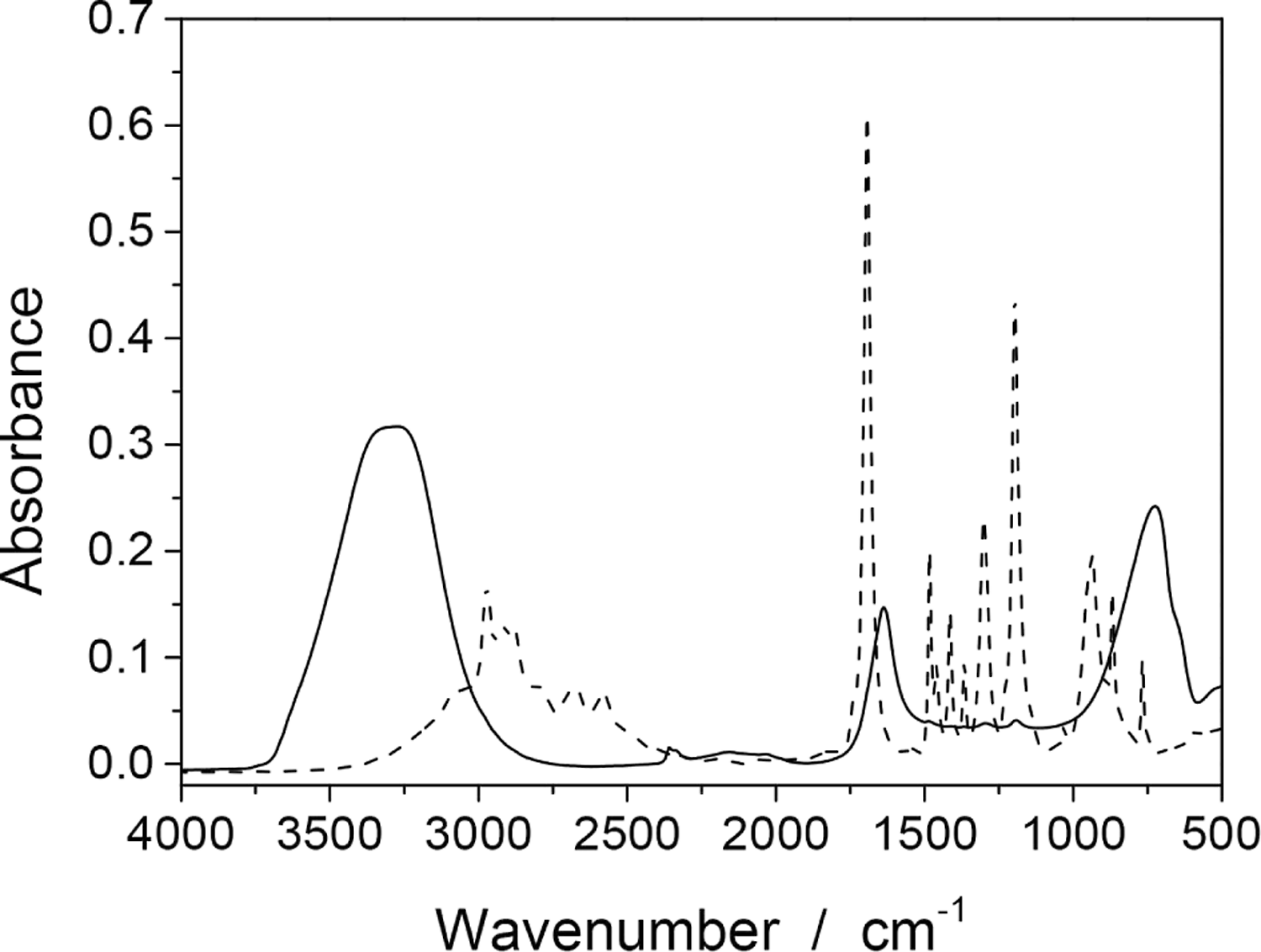
Absorbance spectra of pivalic acid and its solution. The dashed line presents the spectrum of pure pivalic acid condensed on the reflection plane of the ATR prism. The solid line shows the spectrum of aqueous solution of 102 mmol L^–1^, which is half the solubility. The spectrometer was purged with N_2_ during the measurements.

Pivalic acid condensed on the prism gave vibrational bands of C–H stretching at 3000–2500 cm^–1^, as shown by the dashed line in Figure 1. Absorption from stretching motions in COOH as well as bending and rocking motions in CH_3_ groups appeared at 1700–800 cm^–1^. In aqueous solutions examined in this study, water-induced absorption showed intense bands at 3700–2800, 1700–1500, and 1100–600 cm^–1^ with a maximum absorbance of 0.3 as shown with the solid line. To identify the vibrations of pivalic acid in the solutions, we should focus on a wavenumber window of 1500–1100 cm^–1^.

**Figure 2:**
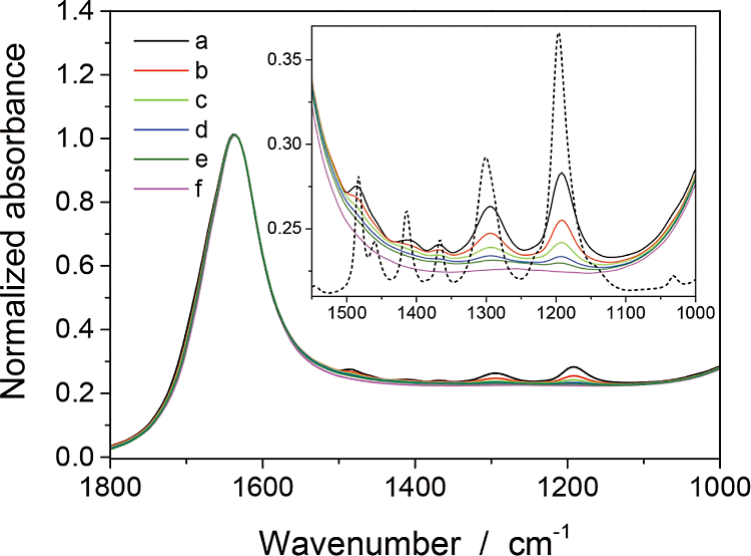
Infrared absorption of six aqueous solutions of pivalic acid. The concentration of pivalic acid was as follows; (a) 102, (b) 51, (c) 26, (d) 13, (e) 6, and (f) 3 mmol L^–1^. Absorbance spectra are normalized to water-induced absorption at 1631 cm^–1^. Normalized spectra in the 1550–1000 cm^–1^ range are shown in the inset, along with the spectrum of condensed pivalic acid (dashed line). The solutions were exposed to air during the measurements.

Figure 2 shows a series of absorption spectra observed in six solutions, which were not suspended with TIO-6 particles. Five absorption bands were identified at 1486, 1411, 1366, 1294, and 1192 cm^–1^ in a solution of 102–6 mmol L^–1^. These five absorption bands corresponded to those of condensed pivalic acid. Two bands at 1461 and 1230 cm^–1^ were present in the spectrum of condensed pivalic acid (dashed line in the inset) but could not be identified in the solutions due to convolution with the 1486 and 1196 cm^–1^ bands, respectively. The absorption bands recognized in Figure 2 are listed in the first two columns of Table 1, along with assignments to vibrational modes based on previous studies.^20,21^ In aqueous solutions, pivalic acid did not show a significant change in vibrational frequency, as the dissociation of pivalic acid to form pivalate anions is limited in water.

**Table 1:** Vibrational Bands Detected in Condensed Pivalic Acid, Its Aqueous Solutions, and Suspensions with TIO-6 Photocatalysts^a^

condensed pivalic acid	solutions (102, 51, 26, 13, and 6 mmol L^–1^)	suspensions (102, 51, and 26 mmol L^–1^)	suspensions (13, 6, and 3 mmol L^–1^)	assignment
1482	1486	1483	1489	CH_3_ bending
1461	not resolved	not resolved	not resolved	CH_3_ bending
1413	1411	1419	1419	OCO symmetric stretching
1364	1366	1369	1369	CH_3_ bending
1300	1294	1292	absent	COH bending
1230	not resolved	1223	1223	CH_3_ rocking
1196	1192	1189	absent	C–OH stretching

a Vibrational wavenumbers are shown in cm^–1^. Pivalic acid concentration is shown in parentheses. Vibrational band assignment based on earlier studies^20,21^ is noted in the rightmost column.

Two absorption bands at 1300 and 1196 cm^–1^ of condensed pivalic acid shifted by 6 and 4 cm^–1^, respectively, to lower wavenumbers in the solutions. These shifts are expected because the bands were associated with atomic motions in the COH group and are, therefore, sensitive to hydrogen bonding with the water solvent.

### 4.2 IR Absorption of Pivalic Acid Adsorbed on TIO-6 Particles

**Figure 3:**
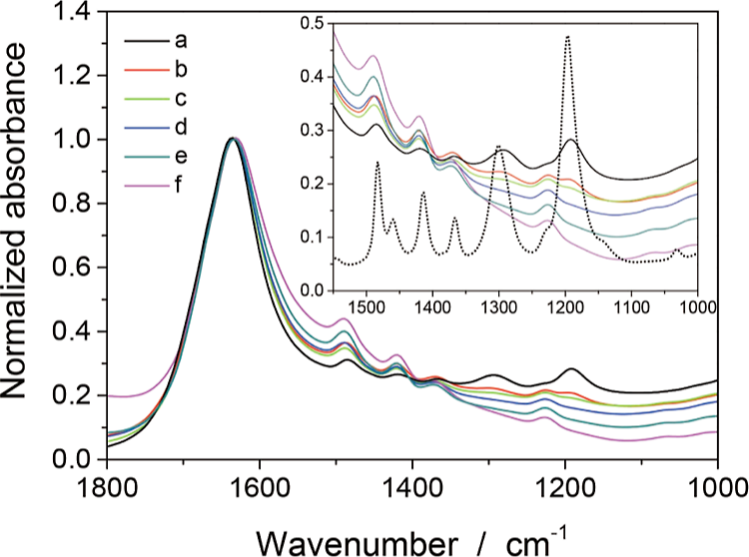
Infrared absorption of TIO-6 photocatalyst when suspended in aqueous solutions of pivalic acid. The concentration of pivalic acid in the solution to be suspended was as follows; (a) 102, (b) 51, (c) 26, (d) 13, (e) 6, and (f) 3 mmol L^–1^. Absorbance spectra were normalized to the water-induced absorption at 1631 cm^–1^. Normalized spectra in the range of 1550–1000 cm^–1^ are shown in the inset, together with the spectrum of condensed pivalic acid (dotted line). The suspensions were exposed to air during the measurements.

Photocatalyst particles (0.10 g) were suspended in the 102 mmol L^–1^ solution of 20 mL and sonicated. Spectrum a in Figure 3 shows the absorbance of the suspension normalized at 1631 cm^–1^. The solution, which was not suspended with TIO-6, showed five vibrational bands. An additional band was detected in the suspension at 1223 cm^–1^ adjacent to the 1189 cm^–1^ band.

A similarly prepared suspension with the 51 mmol L^–1^ solution showed spectrum b, in which two bands at 1292 and 1189 cm^–1^ decreased in intensity while the other bands remained constant. These two bands were further attenuated in spectrum c obtained with a 26 mmol L^–1^ suspension. In suspensions with a concentration of 13 mmol L^–1^ or less, the 1292 and 1189 cm^–1^ bands disappeared, as shown in spectra d–f. The absorption bands recognized in Figure 3 are listed in the middle columns of Table 1.

The systematic variation of the vibrational bands indicated the presence of two chemical species in the suspensions. One species, characterized by absorptions at 1489, 1419, 1369, and 1223 cm^–1^, dominated in suspensions prepared with dilute solutions (13, 6, and 3 mmol L^–1^). This species is attributed to pivalate anions adsorbed on TIO-6 particles because vibrational modes related to undissociated COOH were absent at 1292 and 1189 cm^–1^. Pivalic acid dissociation was negligible even in the dilute solutions, as shown in spectra in Figure 2. Thus, the pivalate anions seen in Figure 3 are likely to be present on TIO-6 particles. The other species was identified in suspensions prepared with concentrated solutions (102, 51, and 26 mmol L^–1^). It is assigned to be molecular pivalic acid because the 1292 and 1189 cm^–1^ bands, characteristic of undissociated COOH, were present. Undissociated pivalic acid was incorporated into the solutions and possibly also onto the TIO-6 particles.

The intensity of the pivalate-induced absorption bands (1489, 1419, 1369, and 1223 cm^–1^) remained constant in the 13, 6, and 3 mmol L^–1^ suspensions. This constant intensity suggested that the TIO-6 particles were saturated with adsorbed pivalate anions. We now estimate the number of pivalate anions on the particles. Supposing that TIO-6 particles were terminated with (110) planes, the most stable truncation of rutile, and that these planes were saturated with pivalate anions, forming a (2 × 1) monolayer of the anions, as demonstrated in vacuum.^17^ The density of the anions in this monolayer is 2.6 × 10^18^ anions m^–2^. The suspension contained TIO-6 of 0.10 g and the specific surface area of the particles was 100 m^2^ g^–1^ according to the Catalysis Society of Japan. Therefore, 2.6 × 10^19^ pivalate anions were adsorbed on the TIO-6 particles. Conversely, there were 4 × 10^19^ pivalic acid molecules in the 3 mmol L^–1^ solution of 20 mL. The number of pivalic acid molecules in the solution to be suspended was in balance with the number of pivalate anions adsorbed in the suspension. The balanced numbers indicated that pivalic acid was selectively adsorbed on TIO-6 particles in the 3 mmol L^–1^ suspension, i.e., the adsorption equilibrium shifted toward adsorption.

### 4.3 UV-Induced Reaction in Aerobic Suspension

**Figure 4:**
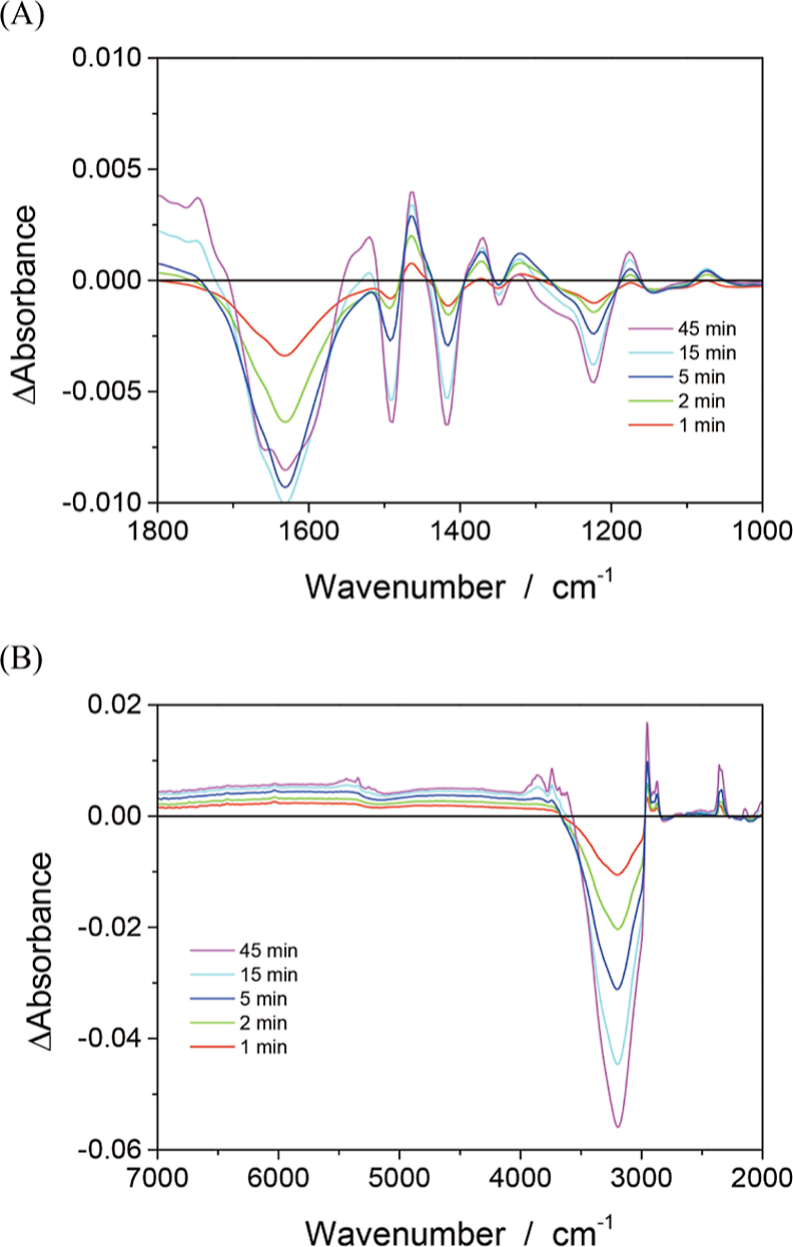
Absorbance change (Δabsorbance) induced by UV light irradiation in air. TIO-6 photocatalyst particles were suspended in aqueous solution of pivalic acid (6 mmol L^–1^) and irradiated with 365 nm light for 1, 2, 5, 15, and 45 min. The power density of the UV light was 3.6 kW m^–2^. Responses in the 1800–1000 and 7000–2000 cm^–1^ ranges are shown in panels (A,B), respectively.

The photocatalytic conversion of adsorbed pivalate anions described in formula 2 was investigated in the suspension prepared with the 6 mmol L^–1^ solution. Undissociated pivalic acid was almost absent in the suspension, as shown by spectrum e in Figure 3. The suspension was placed on the prism, exposed to air, and irradiated with UV light through the prism. The power density of the UV light was calibrated to be 3.6 kW m^–2^ at the reflection plane using a photodiode sensor (PD-300, Ophir). Figure 4A shows the response (Δabsorbance) of the pivalate-induced absorption bands. The background level of Δabsorbance spectra gradually shifted due to the slow evaporation of liquid N_2_ in the Dewar bottle of the MCT detector. To compensate for these background shifts, the Δabsorbance spectra were set to zero at 1000 cm^–1^.

During the first 15 min of UV irradiation, the absorbance at 1492, 1415, 1349, 1222 cm^–1^ decreased and reached saturation after 45 min of irradiation. The decreased absorbance indicated adsorbed pivalate anions that were converted under irradiation. Vibrational bands at these wavenumbers were assigned to CH_3_- and OCO-related motions of the anions (Table 1). A broad, negative band appeared at 1620 cm^–1^, which was assigned to water-bending absorption bleached under irradiation. On the other hand, the absorbance at 1465, 1370, 1176, and 1074 cm^–1^ increased. In a transient IR absorption study conducted in vacuum,^20^ pivalate anions adsorbed on platinum-loaded TiO_2_ particles showed increased absorbance at 1477 and 1372 cm^–1^ when irradiated with UV light pulses. Of the increased absorbance observed in Figure 4A, the increments at 1465 and 1370 cm^–1^ are consistent with those reported in the vacuum. Absorbance increments at these two wavenumbers are assigned to bending motions of CH_2_ or CH_3_ in the end products of the photocatalytic reaction, isobutene and isobutane, produced via *t*-butyl radical intermediates. The assignment of the two bands at 1176 and 1074 cm^–1^ remains unknown.

Figure 4B shows the response in the 7000–2000 cm^–1^ region. There are significant responses at 3000–2900 and 2350 cm^–1^. The peaks at 3000–2900 cm^–1^ can be attributed to C–H stretching enhanced by isobutene and isobutane production. The increased absorbance at 2350 cm^–1^ indicates the release of CO_2_ during the hole-induced decarboxylation of pivalate anions. The decreased absorbance in the 3500–3000 cm^–1^ region is due to OH stretching of water bleached by UV irradiation.

### 4.4 UV-Induced Reaction in Anaerobic Suspension

**Figure 5:**
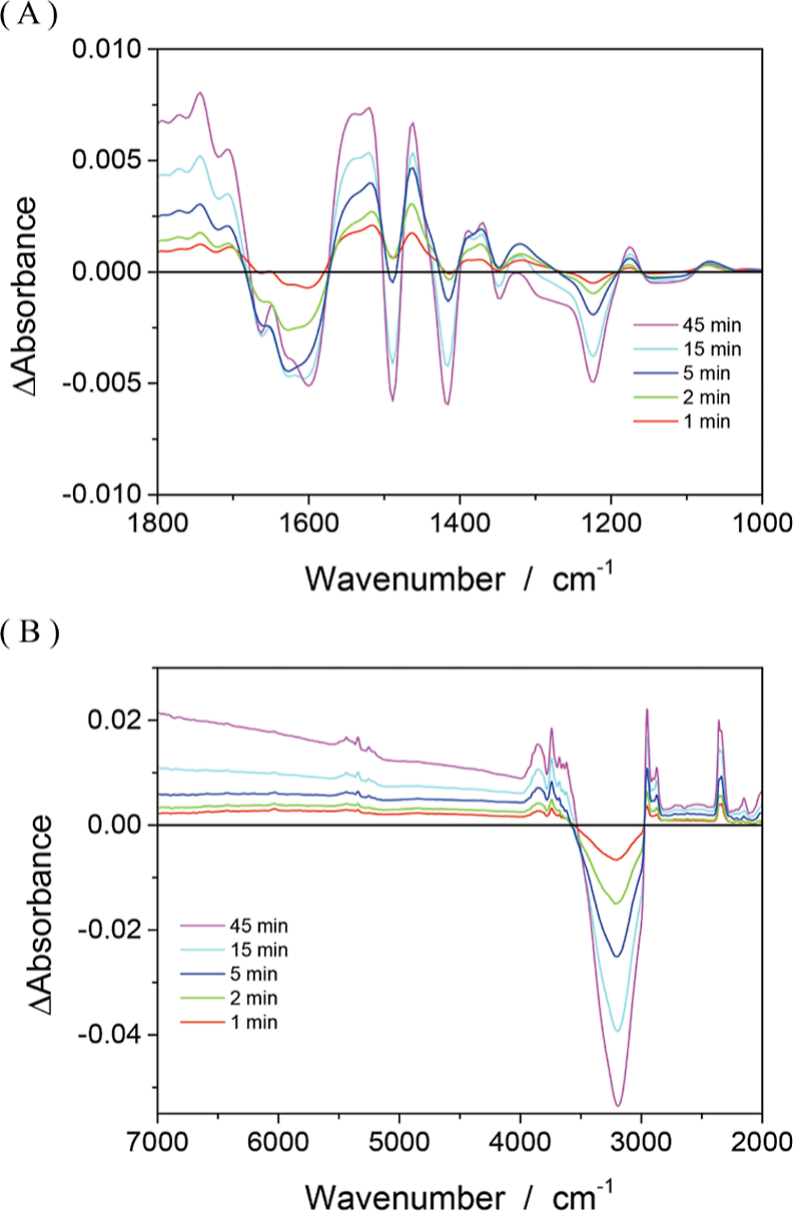
Absorbance change (Δabsorbance) induced by UV light irradiation in a N_2_ atmosphere. TIO-6 photocatalyst particles were suspended in aqueous solution of pivalic acid (6 mmol L^–1^) and irradiated with 365 nm light for 1, 2, 5, 15, and 45 min. The power density of the UV light was 3.6 kW m^–2^. Responses in the 1800–1000 and 7000–2000 cm^–1^ ranges are shown in panels (A,B), respectively. The background of the Δabsorbance spectra shifted due to the evaporation of liquid N_2_ in the MCT detector. To compensate for these background shifts, the Δabsorbance spectra were set to zero at 1000 cm^–1^.

Finally, we compared the UV-induced response in an anaerobic suspension with that in the aerobic suspension at the same concentration (6 mmol L^–1^). The Δabsorbance spectra depicted in Figure 5A,B show that the pivalate-induced and water-induced bands were bleached. Absorption by CH stretching at 3000–2900 cm^–1^ and CO_2_ stretching at 2350 cm^–1^ was simultaneously enhanced. These negative and positive responses are consistent with those observed in the suspension exposed to air.

An additional response appeared under the N_2_ atmosphere. UV irradiation progressively produced a broad absorption, rising at 4000 cm^–1^, and centered at 7000 cm^–1^. Our previous study^8^ of TIO-6 irradiated under a methanol–water mixture showed that electrons excited across the band gap formed small polarons, which was characterized by optical absorption spread over the 10,000–700 cm^–1^ range with a maximum at 6000 cm^–1^. The broad absorption visible in Figure 5B, although interrupted by vibrational absorption bands in the middle, provides evidence of excited electrons accommodated in TIO-6 particles under the anaerobic suspension.

Semiconductor photocatalysts tend to be reduced and poisoned when excited electrons remain in the semiconductor particles.^22^ This is the case here. Excitation across the band gap creates an electron and a hole. An adsorbed pivalate anion receives a hole, resulting in its decarboxylation. When oxygen is present in the environment, complementary electrons are removed from the TiO_2_ particle to produce water, as formulated in (3). In the absence of oxygen, the electrons are trapped on surface Ti cations, as in formula 4. Hole-induced decarboxylation of pivalate is suspended as holes recombine with the trapped electrons before attacking pivalate anions. Hole-induced decarboxylation should be coupled with electron-induced water production to steadily convert pivalic acid over rutile photocatalysts. This set of findings has been established on (110)-oriented rutile wafers placed in vapor environments, using mass spectrometry of photodesorbed products^16^ and scanning tunneling microscopy of surface chemical species.^17^ Optical absorption spectra shown in Figures 4 and 5 suggest a common chemistry in the photocatalytic conversion of pivalate under both vapor and water environments.

## 5 Conclusions

This study presents the characterization of rutile particles suspended in aqueous solutions of pivalic acid using IR absorption. The diamond prism, used for total reflection of IR light, provided a convenient and reliable method to study the IR absorption spectrum of suspensions under UV light irradiation. When rutile particles were introduced into the solutions, the vibrational bands associated with the COH group of pivalic acid disappeared, indicating the dissociative adsorption of pivalic acid onto the particles. The adsorbed pivalate anions were found to capture photoexcited holes, releasing CO_2_ and hydrocarbons. In the suspension exposed to an N_2_ atmosphere, the excited electrons remained within the particles as small polarons, exhibiting optical absorption centered at 7000 cm^–1^. In contrast, when the suspension was exposed to air, the electrons were consumed to produce water, preventing the presence of polaron-induced absorption. Overall, this research demonstrates the feasibility of the ATR-based IR spectroscopy for the operando characterization of photochemical reactions at liquid–solid interfaces. This was achieved by simultaneously monitoring vibrational and electronic transitions.
